# Alterations in Functional and Structural Connectivity in Pediatric-Onset Multiple Sclerosis

**DOI:** 10.1371/journal.pone.0145906

**Published:** 2016-01-05

**Authors:** Nadine Akbar, Antonio Giorgio, Christine Till, John G. Sled, Sam M. Doesburg, Nicola De Stefano, Brenda Banwell

**Affiliations:** 1 Neurosciences and Mental Health Program, Research Institute, The Hospital for Sick Children, Toronto, Ontario, Canada; 2 Institute of Medical Science, University of Toronto, Toronto, Ontario, Canada; 3 Department of Medicine, Surgery and Neuroscience, University of Siena, Siena, Italy; 4 Department of Psychology, York University, Toronto, Ontario, Canada; 5 Department of Medical Biophysics, University of Toronto, Toronto, Ontario, Canada; 6 Mouse Imaging Centre, The Hospital for Sick Children, Toronto, Ontario, Canada; 7 Department of Neurology, Children’s Hospital of Philadelphia, Philadelphia, Pennsylvania, United States; University of North Carolina, UNITED STATES

## Abstract

**Background:**

Reduced white matter (WM) integrity is a fundamental aspect of pediatric multiple sclerosis (MS), though relations to resting-state functional MRI (fMRI) connectivity remain unknown. The objective of this study was to relate diffusion-tensor imaging (DTI) measures of WM microstructural integrity to resting-state network (RSN) functional connectivity in pediatric-onset MS to test the hypothesis that abnormalities in RSN reflects changes in structural integrity.

**Methods:**

This study enrolled 19 patients with pediatric-onset MS (mean age = 19, range 13–24 years, 14 female, mean disease duration = 65 months, mean age of disease onset = 13 years) and 16 age- and sex-matched healthy controls (HC). All subjects underwent 3.0T anatomical and functional MRI which included DTI and resting-state acquisitions. DTI processing was performed using Tract-Based Spatial Statistics (TBSS). RSNs were identified using Independent Components Analysis, and a dual regression technique was used to detect between-group differences in the functional connectivity of RSNs. Correlations were investigated between DTI measures and RSN connectivity.

**Results:**

Lower fractional anisotropy (FA) was observed in the pediatric-onset MS group compared to HC group within the entire WM skeleton, and particularly the corpus callosum, posterior thalamic radiation, corona radiata and sagittal stratum (all p < .01, corrected). Relative to HCs, MS patients showed higher functional connectivity involving the anterior cingulate cortex and right precuneus of the default-mode network, as well as involving the anterior cingulate cortex and left middle frontal gyrus of the frontoparietal network (all p < .005 uncorrected, k≥30 voxels). Higher functional connectivity of the right precuneus within the default-mode network was associated with lower FA of the entire WM skeleton (r = -.525, p = .02), genu of the corpus callosum (r = -.553, p = .014), and left (r = -.467, p = .044) and right (r = -.615, p = .005) sagittal stratum.

**Conclusions:**

Loss of WM microstructural integrity is associated with increased resting-state functional connectivity in pediatric MS, which may reflect a diffuse and potentially compensatory activation early in MS.

## Introduction

Multiple sclerosis (MS) is an inflammatory demyelinating and neurodegenerative disease of the central nervous system associated with diffuse damage to white matter (WM) tracts. Approximately 5% of all MS patients experience pediatric-onset MS [1–3]. Pediatric MS patients may be especially vulnerable to disruption of WM pathways given that MS onset occurs during the primary maturation of these networks.

Diffusion tensor imaging (DTI) is a technique that characterizes microstructural tissue integrity based on properties of diffusion, and this information is represented mathematically in a diffusion ellipsoid [4, 5]. The diffusion ellipsoid allows for the calculation of fractional anisotropy (FA), axial diffusivity (AD), and radial diffusivity (RD). FA is a measure which incorporates both AD and RD and represents the preferential directionality of water diffusion, often evaluated specifically in WM tracts [4, 5]. AD represents diffusivity parallel to the main axis of WM tracts and higher values are considered to be indicative of axonal loss [6, 7]. RD represents diffusivity perpendicular to the main axis and higher values are more related to demyelination [8]. Collectively, lower FA values are indicative of decreased WM microstructural integrity.

DTI studies have demonstrated widespread abnormalities in pediatric MS patients [9–14]. Reduced FA has been noted not only in T_2_-hyperintense lesions, but also in normal-appearing, non-lesional WM [15]. In addition, average FA of normal-appearing WM shows significant correlations with total lesion load [16]. Higher gray matter mean diffusivity has also been reported in pediatric MS [11]. DTI abnormalities, particularly lower FA of the corpus callosum, appear clinically relevant given associations with reduced attentional control [17], slower cognitive processing speed [18], and poorer math performance [19].

Studies in adults with MS have demonstrated that greater WM disruptions (as measured by DTI) correspond to reduced functional connectivity of corresponding resting-state networks [20, 21], supporting a direct relationship between anatomical and functional connectivity. It has yet to be determined fully whether such a relationship exists in pediatric MS.

Given that pediatric-onset MS patients have a longer time from onset to progressive physical disability relative to adult-onset MS patients [22], it is possible that onset of MS during childhood and adolescence is associated with adaptive resilience that serves to promote functional preservation. In support of the concept of greater functional reserve in pediatric-onset MS, the study by Rocca and colleagues [23] demonstrated that lower FA in the normal appearing WM of the corpus callosum was associated with increases (rather than decreases) in connectivity between the right cerebellum and the left primary sensorimotor cortex during the performance of a simple motor task.

We explored functional connectivity within resting state networks and relate these findings to DTI measures of WM microstructure in a cohort of patients with pediatric-onset MS. We anticipate that pediatric MS patients will demonstrate reduced WM integrity, as measured by DTI. In contrast to adult MS patients, we hypothesize that this loss of WM integrity will promote an early, age-related compensatory increase in resting-state functional connectivity.

## Methods

### Participants

We recruited 23 pediatric-onset MS patients (age less than 18 years at time of first MS attack, all meeting 2010 McDonald criteria [24]) and 20 age- and sex-matched healthy controls. Four MS patients and three controls were excluded due to excessive motion on MRI resulting in a final sample size of 19 pediatric-onset MS and 16 healthy controls. MS patients were recruited from the pediatric (The Hospital for Sick Children) MS program in Toronto, and were evaluated for the present study at York University. Healthy controls were recruited through advertisement. The research protocol was reviewed and approved by the Hospital for Sick Children Research Ethics Board and York University Research Ethics Board (Human Participants Review Committee). Written, informed consent was obtained from all participants and/or a parent or legal guardian. Participants were excluded if they endorsed a history of head trauma, alcohol abuse, illicit drug use, had visual or motor difficulties that would preclude testing, or any other major medical illness. Controls were also required to be free of any neurological illness, previous or current mood disorder, or known learning disability. MS participants were evaluated at least four weeks from clinical relapse or corticosteroid treatment. For each participant, the study took place in one 4-hour session consisting of questionnaires, neuropsychological assessment, and the MRI scan.

### Measures

Demographic and disease-related information were obtained from clinical records. The Expanded Disability Status Scale (EDSS) score [25] within the last six months, relapse history, disease duration, and medications were recorded. The following questionnaires were also administered to all participants in order to compare to two groups on these measures: (a) Dutch Handedness Questionnaire [26], (b) Centre for Epidemiological Studies Depression Scale for Children (CES-DC) [27], (c) Pediatric Quality of Life Inventory Multidimensional Fatigue Scale (PedsQL) [28] and (d) Barratt Simplified Measure of Social Status (BSMSS) [29].

### MRI protocol

Data were acquired on a Siemens MAGNETOM 3T Tim Trio MRI scanner at York University with a 32-channel head coil. The entire scanning protocol (90 minutes) comprised of T_1_-weighted sagittal MPRAGE, resting-state fMRI, three task-based fMRI paradigms, DTI, proton-density (PD), T_2_-weighted, and FLAIR sequences. Sagittal high-resolution three-dimensional (3D) MPRAGE T_1_-weighted images were acquired (TR = 2300ms, TE = 2.96ms, field of view (FOV) = 256x240x192mm, number of slices = 192, voxel size = 1.0x1.0x1.0mm) for the purposes of brain volume measurements, image registration and anatomical mapping. DTI images were recorded using a 2D echo-planar imaging (EPI) sequence with diffusion weighting in 64 directions and b-value of 1000s/mm^2^ (TR = 4600 ms, TE = 93ms, FOV = 256x256x108mm, number of slices = 36, voxel size = 2.0x2.0x3.0mm). Resting-state fMRI images were recorded using a gradient-echo EPI sequence. The duration was six minutes (180 volumes, TR = 2000ms) and parameters included: TE = 30ms, voxel size = 3.0x3.0x4.0mm3, matrix size = 63x85x34, 34 axial slices, flip angle = 90°. Pulse oximetry and respiration were not recorded. During the resting-state sequence participants were instructed to keep their eyes closed, remain motionless, stay awake, and not to think of anything in particular. The PD, T_2_-weighted, and FLAIR acquisitions were used to define T_2_-lesion volumes according to previously published methods [30]. Global and regional brain volumes were obtained using the T_1_-weighted images also according to previously established methods [31]. Normalized thalamic volumes were calculated by dividing absolute thalamic volume by total brain volume.

### Diffusion Tensor Imaging processing

DTI processing was conducted using FMRIB Software Library (FSL) software library tools (www.fmrib.ox.ac.uk/fsl/) [32]. First, DTI data were corrected for MRI eddy currents and head motion using affine registration to a reference volume, i.e., the one without diffusion weighting (b = 0). Then, images were brain-extracted using Brain Extraction Tool (BET) [33] and entered into the program DTIFIT which fits a diffusion tensor model at each voxel. Images were created for each subject representing FA, AD (*λ*_1_), RD [(λ_2_ + λ_3_)/2)] on a voxel-wise level. Tract-Based Spatial Statistics (TBSS) analysis [34] created a nonlinear registration of all FA images to a 1x1x1mm^3^ standard space (FMRIB58_FA), followed by creation of a mean FA image which was further refined to create a mean FA WM skeleton thresholded at a FA value of 0.2. As all subject’s FA images were in a standard space, the WM skeleton was applied yielding FA skeletonized images for each subject. The same WM skeleton was applied to the AD and RD images. For voxelwise analyses of DTI measures (FA, AD, RD), differences between groups were tested in a general linear model (GLM) framework with unpaired t-tests using nonparametric permutation testing (number of permutations = 5000), FSL’s *randomise* [35] and controlling for age and sex. The generated spatial maps characterizing between-group differences were controlled for multiple comparisons using threshold-free cluster enhancement, a method in which voxelwise p-values incorporate the amount of cluster-like local spatial support [36]. The generated p-value maps were thresholded at p = .05.

### Functional MRI data pre-processing

Functional MRI data preprocessing was conducted using a mixture of Analysis of Functional NeuroImages (AFNI) [37] and FSL software library tools. The preprocessing steps included: (a) removal of the first four volumes; (b) motion correction by realigning via rigid transformation to the first volume (scans with a maximum displacement of more than 3mm were discarded); (c) removal of skull and non-brain tissue using FSL's BET; (d) spatial smoothing at a Gaussian kernel of 5-mm full width at half maximum; (e) spatial transformation to ICBM152 template space [38] and resampling to 3mm^3^ voxels. The transformation was done using a combination of 12-parameter affine transformation and nonlinear registration tools and incorporating the T1-weighted image; (f) regression of the functional data against six motion parameter timeseries as well as four WM and four cerebrospinal fluid voxel timeseries in order to remove non-neural contributions to the BOLD signal; and (g) temporal filtering in order to retain frequencies between the range of 0.01 to 0.1 Hz.

### Independent Components Analysis (ICA)

The functional connectivity analysis utilized an ICA-based approach (using FSL’s MELODIC, Multivariate Exploratory Linear Decomposition into Independent Components) in combination with a dual regression technique [39]. The pre-processed fMRI data, which contained 176 time points for each subject, were temporally concatenated across all subjects to create a single 4-dimensional dataset. This fMRI dataset was then decomposed into independent components where the number of dimensions was automatically estimated using the Laplace approximation to the Bayesian evidence of the model order [40]. Outputted components that were noise-related (e.g. due to head motion, cardiac, respiratory, and CSF pulsations), scanner-related artifacts, and misregistrations were discarded before further analysis. For each retained component (i.e. components considered meaningful and corresponding to canonical resting-state networks), dual regression was used to create subject-specific versions of these components [39]. For each ICA component (at the group or individual-level), the voxel-wise intensity values on these spatial maps are parameter estimates (converted to z-scores) representing the amount of co-activation or synchronization of those voxels with the particular component/network and referred to throughout this manuscript as “functional connectivity”. We then tested for between-group differences in parameter estimates using the same FSL *randomise* [35] nonparametric permutation testing tool as described above for the DTI processing. Here, because no clusters survived correction for multiple comparisons at p<0.05, an uncorrected p-value threshold of 0.005 was selected with an arbitrary cluster threshold of >30 voxels [41]. Clusters containing voxels spatially within or touching the network were considered significant. Subsequently, in order to further confirm our results, we computed within significant clusters mean values across all voxels for each subject and performed between-group comparisons with analysis of variance, with Bonferonni correction for multiple comparisons. These mean values were also used to correlate with the values of DTI measures from significant clusters.

## Results

Demographic, disease-related, and structural MRI data are reported in Table 1. As reported in Table 1 there were no differences between groups with respect to age, sex, socioeconomic status, depression, fatigue, or handedness. Lower thalamic volumes were observed in the pediatric-onset MS group compared to healthy control group. There were also no differences between groups with respect to motion on either the DTI or resting-state fMRI scans based on maximum displacement (in any direction, mm) relative to first volume (Table 1).

**Table 1:** Demographic, Disease-Related, and Structural MRI Data for all Subjects.

Variable		Pediatric-onset MS (n = 19)	Healthy Controls (n = 16)	Between-groups comparison (t-test or X^2^)	p-value
**Mean (range) age at scan, years**		18.7 (13–24)	19.1 (13–24)	.403	.69
**Sex (F/M)**		14/5	11/5	.104	.748
**Mean (SD) disease duration**^**a**^**, months**		64.5 (37.8)			
**Mean (SD) age at MS onset**^**b**^**, years**		13.2 (2.72)			
**Median (range) EDSS score**		1.5 (0–4)			
**Median (range) total number of relapses since MS onset**		4 (1–13)			
**Current use of disease-modifying therapies (Y/N)**		17/2			
**Mean (SD) Socioeconomic status- BSMSS score**		44.3 (13.8)	39.0 (15.4)	-1.04	.307
**Mean (SD) Depression- CES-DC score**		14.9 (13.1)	11.9(8.0)	-.801	.429
**Mean (SD) Fatigue- PedsQL Multidimensional Fatigue score**		30.0 (13.4)	23.3(8.8)	-1.677	.103
**Handedness (Right/Left)**^**c**^		16/3	15/1	1.756	.416
**Mean (SD) Structural MRI metrics**					
	**T**_**2**_**-Lesion Volume (cm**^**3**^**)**	14.1 (26.3)			
	**T**_**1**_**-Lesion Volume (cm**^**3**^**)**	8.94 (18.2)			
	**Brain Volume (cm**^**3**^**)**	1328 (127.9)	1296 (133.8)	-.722	.476
	**Thalamic Volume (cm**^**3**^**)**	11.64 (1.39)	12.65 (1.078)	2.38	.023*
	**Normalized thalamic volume**^**d**^	.00880 (.00090)	.00978 (.00035)	4.10	< .001**
**Mean (SD) motion (maximum displacement in mm)**					
	**DTI**	2.23 (1.67)	2.27 (1.10)	.067	.947
	**Resting-state fMRI**	1.06 (.774)	1.42 (.672)	1.46	.153

EDSS = Expanded Disability Status Scale; BSMSS = Barratt Simplified Measure of Social Status (BSMSS); CES-DC = Centre for Epidemiological Studies Depression Scale for Children; PedsQL = Pediatric Quality of Life Inventory Multidimensional Fatigue Scale.

^a^Months since first attack

^b^Age at first attack

^c^Based on Dutch Handedness Questionnaire

^d^Normalized thalamic volume = thalamic volume/ brain volume

*p < .05

**p < .01

### Correlation between DTI and demographic and disease-related variables

Within the MS group, none of the DTI metrics showed a significant association with age, sex, age of disease onset, disease duration, or EDSS. All FA, AD, and RD measures correlated significantly with T_2_- lesion volume (FA: r = -.782, p < .001; AD: r = .684, p = .001, and RD: r = .788, p < .001 for correlations with the whole WM skeleton) as well as with thalamic volumes (FA: r = .707, p = .001; AD: r = -.782, p < .001; RD: r = -.806, p < .001 for correlations with the whole WM skeleton).

### Between-group DTI differences

**Fig 1:**
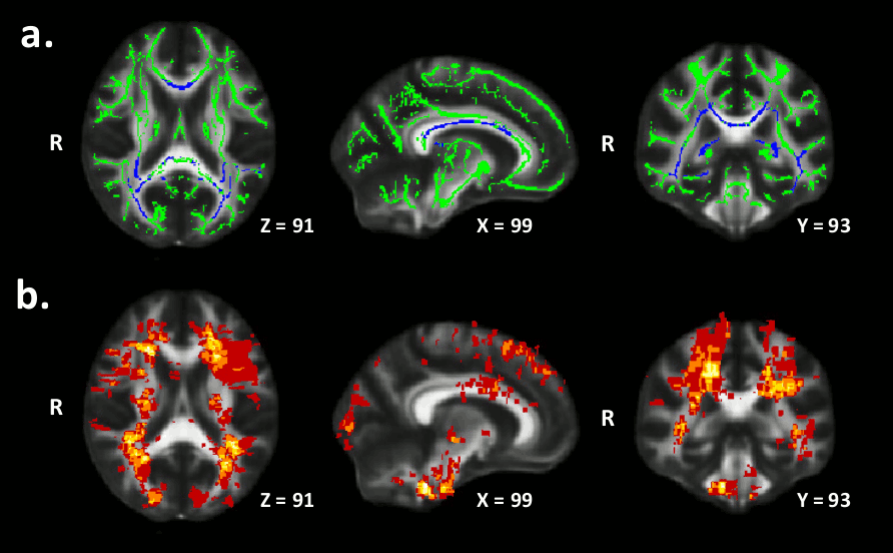
Differences between groups in white matter FA. (a) WM skeleton (green) depicting areas in which the pediatric-onset MS group demonstrated lower FA (blue) compared to the healthy control group (p < .01, corrected). (b) Mean lesion map of the pediatric MS group with brighter (yellow) areas representing voxels with higher probability of lesion occurrence. MNI152 template slice coordinates are also reported.

Pediatric-onset MS patients demonstrated lower WM FA compared to healthy controls of the entire WM skeleton (t = 2.84, p = .008, reported in Table 2). Areas demonstrating the largest differences between groups were the corpus callosum, posterior thalamic radiation, sagittal stratum (including the inferior longitudinal fasciculus and inferior fronto-occipital fasciculus), and corona radiata (Fig 1A, Table 2). The pediatric-onset MS group also demonstrated higher entire WM skeleton RD, but no difference in AD compared to the healthy control group (Table 2). Voxel-wise differences between groups in WM RD and AD are shown in S1 Fig. There were no brain regions in which the healthy controls showed lower FA or higher diffusivity relative to the MS patients.

**Table 2:** Differences Between Groups on DTI and fMRI Measures.

Measure (mean, SD)		Pediatric MS (n = 19)	Healthy Control (n = 16)	t-test	p-value
**Fractional Anisotropy**					
	**Entire WM Skeleton**	.437 (.025)	.458 (.015)	2.84	.008**
	**Within Significant FA voxels**^**a**^	.551 (.041)	.616 (.020)	5.84	< .001**
	**Corpus Callosum- Body**^**b**^	.637 (.058)	.714 (.032)	5.02	< .001**
	**Corpus Callosum- Genu**^**b**^	.644 (.065)	.701 (.040)	3.05	.005**
	**Corpus Callosum- Splenium**^**b**^	.748 (.049)	.796 (.016)	4.63	< .001**
	**Posterior Thalamic Radiation- Left**^**b**^	.596 (.053)	.669 (.027)	5.26	< .001**
	**Posterior Thalamic Radiation- Right**^**b**^	.601 (.043)	.680 (.024)	6.50	< .001**
	**Corona Radiata- Left Superior**^**b**^	.445 (.047)	.497 (.029)	4.03	< .001**
	**Corona Radiata- Right Superior**^**b**^	.443 (.046)	.493 (.026)	4.02	< .001**
	**Corona Radiata- Left Posterior**^**b**^	.485 (.050)	.542 (.029)	4.15	< .001**
	**Corona Radiata- Right Posterior**^**b**^	.463 (.047)	.522 (.021)	4.91	< .001**
	**Sagittal Stratum- Left**^**b**^	.536 (.041)	.594 (.023)	4.75	< .001**
	**Sagittal Stratum- Right**^**b**^	.536 (.045)	.607 (.028)	5.44	< .001**
**Axial Diffusivity (10**^**-3**^**mm**^**2**^**/sec)**					
	**Entire WM Skeleton**	1.26 (.042)	1.24 (.023)	-1.43	.163
	**Within Significant FA voxels**^**a**^	1.53 (.057)	1.51 (.029)	-1.07	.294
**Radial Diffusivity (10**^**-3**^**mm**^**2**^**/sec)**					
	**Entire WM Skeleton**	.622 (.052)	.585 (.027)	-2.51	.017*
	**Within Significant FA voxels**^**a**^	.534 (.013)	.433 (.049)	-3.22	.004**
**Functional connectivity (z-scores)**^**c**^					
	**Bilateral frontoparietal network- C1, Anterior cingulate cortex**	3.20 (2.34)	-2.67 (3.08)	-6.40	< .001 **
	**Bilateral frontoparietal network- C2, Left middle frontal gyrus**	11.92 (5.75)	3.43 (4.64)	-4.74	< .001**
	**Precuneus network- C3, Right precuneus**	9.51 (5.74)	1.49 (4.42)	-4.56	< .001**
	**Precuneus network- C4, Anterior cingulate cortex**	3.21 (4.66)	-5.77 (4.83)	-5.59	< .001**

^a^ mean values reported are for only those voxels that demonstrated significantly different FA values between groups in t-test analysis

^b^ mean values reported are for only those voxels that demonstrated significantly different FA values between groups in t-test analysis, restricted to certain WM structures as defined by FSL’s JHU white-matter tractography atlas (Mori et al. 2008)

^c^mean values reported are for clusters depicted in Fig 2K. These clusters represent only those areas that differed significantly between groups in the voxel-wise dual regression analysis.

*p < .05

**p < .01

### Resting state networks

**Fig 2:**
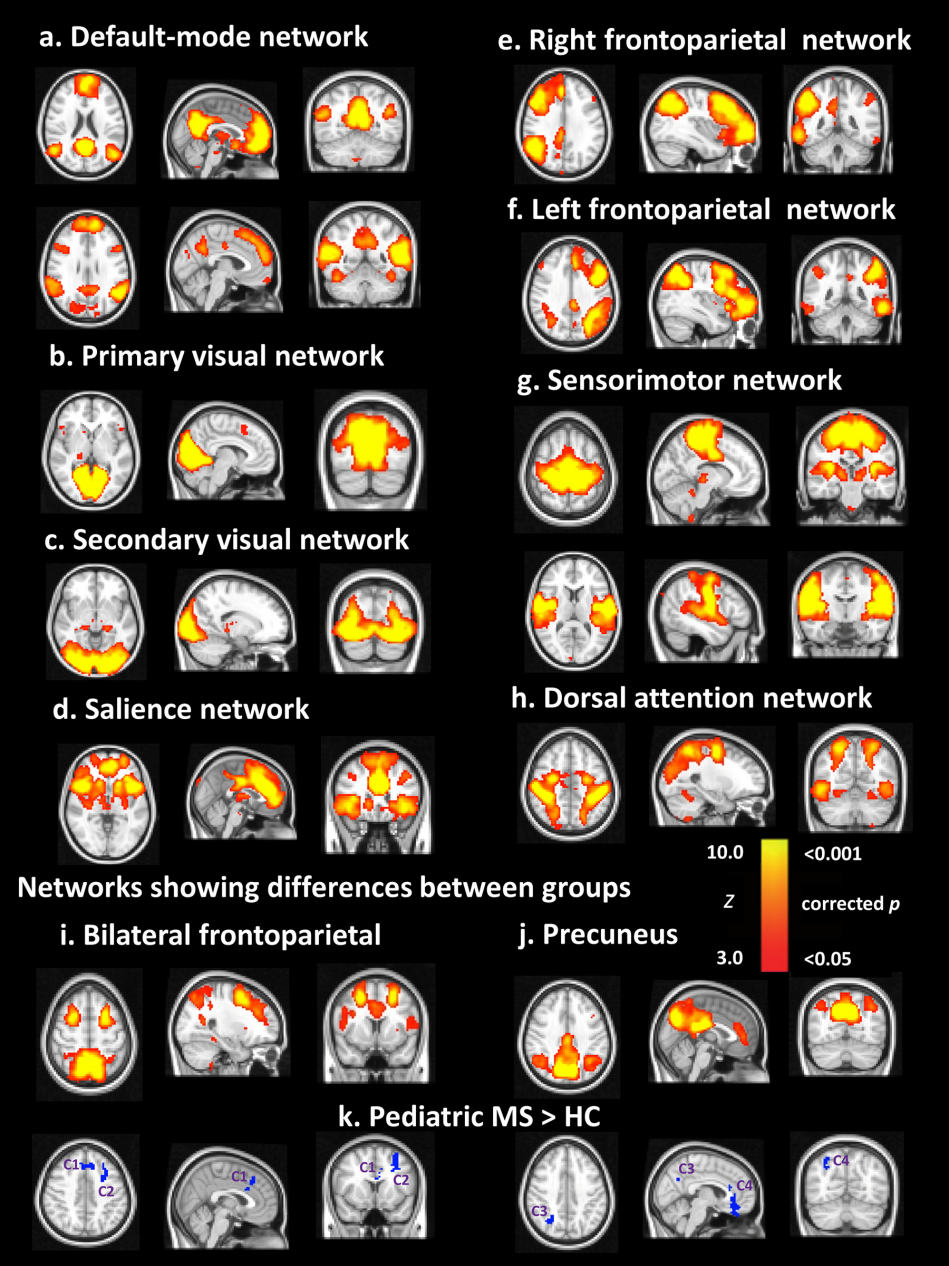
Differences between groups in functional connectivity of resting-state networks. Group ICA components (3D volumes) including (a) default mode network, (b) primary visual network, (c) secondary visual network, (d) salience network, (e) right frontoparietal network, (f) left frontoparietal network, (g) sensorimotor network, and (h) dorsal attention network. The ICA components are shown in FSL red-yellow encoding using a 3< z-score <10 threshold. The (i) bilateral frontoparietal and (j) precuneus (posterior default-mode) networks were the only networks which demonstrated significant differences between groups (p < .005 uncorrected, cluster size> 30 voxels). Areas in blue below these networks (k), with numbered clusters 1–4, indicate those areas within these networks in which the pediatric-onset MS group demonstrated higher connectivity compared to healthy controls. C1 indicates the anterior cingulate cluster and C2 the left middle frontal gyrus cluster of the bilateral frontoparietal network. C3 indicates the right precuneus and C4 the anterior cingulate cluster of the precuneus posterior default-mode network. Statistics for the connectivity values of these clusters are referred to throughout the text. All images are displayed in radiological convention. (Left = Right, Right = Left). The most informative slices are shown.

Group ICA of all 35 subjects revealed eight canonical resting-state networks (Fig 2) out of the 19 components that were outputted from ICA (n = 11 were discarded as noise/artifact related or misregistration). These networks are a replication of commonly reported resting-state networks and were labelled accordingly [42–44]. These networks included the: (a) default-mode network (3 components, including precuneus in Fig 2J), (b) primary and (c) secondary visual networks, (d) salience network, (e) frontoparietal network (separate right and left components, and one bilateral component, Fig 2I), (f) sensorimotor network (2 components), and (g) dorsal attention network. Overall, there were two components in which the pediatric-onset MS group demonstrated higher functional connectivity than the healthy control group (Fig 2K). These two components comprised the frontoparietal network bilaterally, and default mode network precuneus. Within the bilateral frontoparietal network, higher functional connectivity was demonstrated at the level of the anterior cingulate cortex and left middle frontal gyrus in the pediatric MS group (statistics reported in Table 2). Within the default mode network precuneus, higher functional connectivity was demonstrated within the anterior cingulate cortex and right precuneus. Healthy controls did not demonstrate higher connectivity relative to the MS patients in any network.

### Correlations between DTI measures and functional connectivity

Higher functional connectivity of the right precuneus within the default mode network (cluster 3, Fig 2K) was associated with lower FA and higher RD in the pediatric-onset MS group (all correlations reported in Table 3). With respect to FA, functional connectivity of the right precuneus within the default mode network was significantly correlated with whole WM skeleton FA (r = -.525, p = .021), and FA of the following structures: (a) genu of corpus callosum (FA: r = -.553, p = .014; RD: r = .679, p = .001), (b) left sagittal stratum (FA: r = -.467, p = .044; RD: r = .588, p = .008), and (c) right sagittal stratum (FA: r = -.615, p = .005; RD: r = .658, p = .002). Widespread significant associations with RD were also found (all reported in Table 3). No significant correlations between functional connectivity and DTI measures were present in the healthy control group.

**Table 3:** Correlation between DTI and Functional Connectivity Measures within Pediatric MS Group.

Measure (mean, SD)		C1- FrontoPar, ACC	C2- FrontoPar, LMidFron	C3- Default mode, RPrecuneus	C4- Default mode, ACC
**Fractional Anisotropy**					
	**Entire WM Skeleton**	-.329 (.169)	-.153 (.532)	-.525 (.021)*	.042(.865)
	**Within Significant FA voxels**^**a**^	-.181 (.459)	.022 (.929)	-.351 (.140)	.035 (.886)
	**Corpus Callosum- Body**^**b**^	-.085 (.729)	.104 (.672)	-.252 (.298)	.009 (.972)
	**Corpus Callosum- Genu**^**b**^	-.296 (.218)	-.083 (.735)	-.553 (.014)*	.028 (.909)
	**Corpus Callosum- Splenium**^**b**^	-.288 (.232)	.105 (.669)	-.409 (.082)	.021 (.932)
	**Posterior Thalamic Radiation- Left**^**b**^	-.120 (.623)	.088 (.720)	-.094 (.703)	.137 (.575)
	**Posterior Thalamic Radiation- Right**^**b**^	-.004 (.986)	.078 (.751)	-.147 (.548)	.022 (.930)
	**Corona Radiata- Left Superior**^**b**^	.033 (.895)	-.054 (.827)	-.136 (.580)	-.022 (.930)
	**Corona Radiata- Right Superior**^**b**^	-.172 (.481)	.103 (.675)	-.152 (.534)	.176 (.472)
	**Corona Radiata- Left Posterior**^**b**^	-.088 (.721)	.046 (.850)	-.429 (.067)	.093 (.706)
	**Corona Radiata- Right Posterior**^**b**^	-.112 (.647)	.170 (.486)	-.348 (.144)	-.054(.826)
	**Sagittal Stratum- Left**^**b**^	-.111 (.651)	-.078 (.751)	-.467 (.044)*	.061 (.804)
	**Sagittal Stratum- Right**^**b**^	-.150 (.539)	.045 (.855)	-.615 (.005)**	.014 (.955)
**Radial Diffusivity (10**^**-3**^**mm**^**2**^**/sec)**					
	**Entire WM Skeleton**	.361 (.129)	.014 (.956)	.661 (.002)**	-.040 (.872)
	**Within Significant FA voxels** ^**a**^	.256 (.289)	-.130 (.597)	.486 (.035)*	-.005 (.983)
	**Corpus Callosum- Body**^**b**^	.176 (.472)	-.145 (.553)	.350 (.141)	.012 (.962)
	**Corpus Callosum- Genu**^**b**^	.318 (.185)	.105 (.669)	.679 (.001)**	-.043 (.862)
	**Corpus Callosum- Splenium**^**b**^	.314 (.191)	-.176 (.472)	.501 (.029)*	-.008 (.973)
	**Posterior Thalamic Radiation- Left**^**b**^	.140 (.568)	-.170 (.486)	.217 (.372)	-.109 (.656)
	**Posterior Thalamic Radiation- Right**^**b**^	.084 (.733)	-.201 (.408)	.341 (.153)	.034 (.891)
	**Corona Radiata- Left Superior**^**b**^	.201 (.408)	.037 (.880)	.399 (.091)	.102 (.677)
	**Corona Radiata- Right Superior**^**b**^	.385 (.104)	-.207 (.394)	.273 (.259)	-.132 (.591)
	**Corona Radiata- Left Posterior**^**b**^	.245 (.313)	-.141 (.566)	.582 (.009)**	-.052 (.831)
	**Corona Radiata- Right Posterior**^**b**^	.227 (.351)	-.176 (.471)	.601 (.007)**	.063 (.798)
	**Sagittal Stratum- Left**^**b**^	.204 (.402)	-.032 (.896)	.588 (.008)**	-.028 (.911)
	**Sagittal Stratum- Right**^**b**^	.147 (.548)	-.086 (.727)	.658 (.002)**	.013 (.957)

Functional connectivity measures correspond to those clusters depicted in Fig 2K and include C1 (anterior cingulate cortex) and C2 (left middle frontal gyrus) of the bilateral frontoparietal network, C3 (right precuneus) and C4 (anterior cingulate cortex) of the precuneus default-mode network. Pearson correlation coefficients (r-values) are reported with their corresponding p-values.

^a^mean values reported are for only those voxels that demonstrated significantly different FA values between groups in t-test analysis.

^b^mean values reported are for only those voxels that demonstrated significantly different FA values between groups in t-test analysis, restricted to certain WM structures as defined by FSL’s JHU white-matter tractography atlas (Mori et al. 2008).

*p < .05

**p < .01

## Discussion

We confirm a widespread loss of WM integrity in pediatric-onset MS as well as increased resting-state functional connectivity in two key resting-state networks (i.e. default mode and frontoparietal). We also show that reduced WM microstructural integrity is associated with higher functional connectivity of the precuneus suggesting particular disruption of the default mode network with WM damage.

The reduced WM FA seen in our cohort is congruent with other reports in pediatric MS [9–15, 18]. The corpus callosum, posterior thalamic radiation, sagittal stratum, and corona radiata were the WM regions most affected. These structures may be more vulnerable in pediatric MS given that they are in close proximity to regions with high lesional probability (e.g. periventricular areas), which are shown in Fig 1B. Several studies of DTI in pediatric MS have demonstrated a prominent impact of MS on the corpus callosum [9, 10, 12, 14], which is likely related to the fact that the corpus callosum represents the major WM structure in the brain. We also found that all of our DTI measures, including FA of the corpus callosum, correlated with T_2_-lesion volume suggesting Wallerian degeneration as a possible substrate for reduced white matter tract integrity.

Higher levels of resting state functional connectivity has similarly been observed in cognitively intact (or minimally impaired) adults with early relapsing-remitting MS [45–47]. Our cohort consisted of largely cognitively intact patients [48]. We speculate that the higher connectivity in our pediatric MS patients contributed to their preservation of cognitive performance. Serial studies, including patients with variable level of cognitive performance, are required in order to determine whether subsequent loss of compensatory connectivity subserves cognitive decline.

Within our pediatric MS cohort, higher default mode network functional connectivity was associated with reduced FA and higher RD of WM. Similar findings of reduced tissue integrity being associated with heightened connectivity of the default mode network during resting state has also been reported in studies of adult MS [46, 49], suggesting increased cortical functional activity may be necessary to overcome structural damage of WM pathways. Conceptually, loss of tissue microstructure is unlikely to lead to improved connectivity of neural networks, but it is possible that loss of tissue integrity leads to loss of inhibition which in turn leads to increased functional connectivity [50, 51]. It is also possible that the brain responds to the reduction in tissue microstructure by increasing connectivity within preferred and perhaps more essential pathways, such as the more prominent resting-state networks including the default mode and frontoparietal networks. No significant correlations between functional connectivity and DTI were demonstrated in the healthy control group, which we speculate to be due to limited variability and lack of abnormal DTI values in this group.

In summary, we show both anatomical DTI and functional resting-state fMRI abnormalities in pediatric MS. More extensive loss of WM integrity was associated with increased default mode network connectivity at the level of the precuneus which could be interpreted to represent a compensatory response in a cohort of MS patients studied both at a younger age and at an early stage of their disease. DTI and functional connectivity analysis in a larger sample of pediatric-onset MS patients with longer disease duration and more variable cognitive impairment will provide further insight into the relationship between functional network characteristics, structural damage, and cognitive performance.

## Supporting Information

S1 Fig Differences Between Groups in White Matter AD and RD. WM skeleton (green) depicting areas in which the pediatric-onset MS group demonstrated higher (blue) (Figure a) AD and (Figure b) RD compared to the healthy control group (p < .01 corrected). MNI152 template slice coordinates are also reported.
(TIF)
